# Differences in Hepatocellular Carcinoma Incidence Trends Across US Census Divisions, 2001 to 2021

**DOI:** 10.3390/cancers17091431

**Published:** 2025-04-24

**Authors:** Itunu O. Sokale, Omar Rosales, Aaron P. Thrift, Hashem B. El-Serag, Elyse Burgess, Abiodun O. Oluyomi

**Affiliations:** 1 Section of Epidemiology and Population Sciences, Department of Medicine, Baylor College of Medicine, Houston, TX 77030, USAoluyomi@bcm.edu (A.O.O.); 2 Dan L. Duncan Comprehensive Cancer Center, Baylor College of Medicine, Houston, TX 77030, USA; 3 Section of Gastroenterology and Hepatology and Clinical Epidemiology and Comparative Effectiveness Program in the Health Services Research, Michael E. DeBakey VA Medical Center, Baylor College of Medicine, Houston, TX 77030, USA; 4 Center for Precision Environmental Health, Baylor College of Medicine, Houston, TX 77030, USA

**Keywords:** hepatocellular carcinoma, liver cancer, temporal trend, US census divisions, racial and ethnic groups

## Abstract

**Background/Objectives:** Hepatocellular carcinoma (HCC) is a leading cause of cancer diagnoses and deaths in the United States. This study serves as an update on secular trends in national HCC incidence rates while exploring potential geographic and racial/ethnic differences across all nine US census divisions. **Methods**: We analyzed HCC incidence data reported to the United States Cancer Statistics (USCS) database from 2001 to 2021 (excluding 2020 data, based on the Centers for Disease Control and Prevention’s cautionary recommendations for COVID-19 pandemic data usage for trend analysis). We performed trend analyses of age-adjusted incidence rates in the US overall, by census divisions, and then stratified by race/ethnicity, using the National Cancer Institute’s Joinpoint Regression Program. **Results**: Between 2001 and 2021, HCC incidence rates increased nationally, with an average annual percentage change (AAPC) of 2.51 (95% confidence interval (CI): 2.32–2.72); however, the rate decreased (annual percentage change (APC) −3.33 (95% CI: −4.78–−1.96) in recent years from 2018 to 2021. Division 1 had the greatest decrease (APC −6.46 (95% CI: −9.62–−3.96) from 2017 to 2021, while rates leveled in Division 6 (East South Central) and Division 7 (West South Central). HCC trends decreased substantially for non-Hispanic Black and Non-Hispanic Asian and Pacific Islander groups in almost all divisions in recent years, but trends were stable, decreased, or increased for other racial/ethnic populations. **Conclusions**: Despite declining national HCC incidence rates, these trends were not uniform across racial/ethnic groups or US census divisions. Race-specific interventions are needed to reduce disparities in HCC incidence in all US census divisions.

## Simple Summary

Hepatocellular carcinoma (HCC) is a leading cause of cancer-related deaths globally. Current data regarding national HCC disparities may mask important place-based differences in trends. The national HCC incidence rate dropped significantly in recent years from 2018 to 2021, suggesting progress. However, the decrease was not uniform across US geographic divisions and racial/ethnic groups, highlighting the need for additional strategic interventions. Interventions with racial/ethnic- and place-specific considerations may offer practical solutions to HCC disparities.

## 1. Introduction

Primary liver cancer, predominantly hepatocellular carcinoma (HCC), is a major public health issue worldwide. It is the sixth most diagnosed cancer, the third leading cause of cancer-related deaths globally, and the sixth cause of cancer deaths in the US [1]. There are long-standing disparities in HCC burden in the U.S. The reasons for these disparities are multifactorial, but likely include varying prevalences between groups of exposures to known risk factors for HCC, including chronic viral hepatitis (hepatitis B virus or hepatitis C virus), tobacco use, heavy alcohol consumption, obesity, diabetes, and metabolic dysfunction-associated steatotic liver disease (MALSD) [1].

HCC incidence rates have been rising in the US since the early 1980s [2], but recent studies with data through 2019 have suggested a plateau or decline in the trends of overall HCC incidence rates during recent years (2015–2019) [3,4,5]. However, another recent study [6] found racial/ethnic disparities in HCC burden and incidence trends nationally, including incidence data from 2020 in the trend analysis. While findings from these earlier studies are important, there are several gaps in the knowledge. First, analyses at the national level may mask important place-based differences in trends. Therefore, understanding how HCC has impacted specific subgroups in different places in the US over the years is critical for identifying intervention priorities and optimizing resources to mitigate the disease burden and reduce disparities. One available option to study the effect of “place” is census divisions, which are defined as geographic areas containing states with similar historical development, demographic characteristics, and economies [7]. Second, the Centers for Disease Control and Prevention (CDC), in their cautionary recommendations [8], stated that due to cancer data reporting disruptions during the COVID-19 pandemic, using 2020 data may distort joinpoint trend analysis results. Recently, the National cancer Institute updated the Joinpoint program with a feature that allows the exclusion of any data point [9]. Thus, our analysis serves as an update on the overall trends in HCC incidence rates and explores potential geographic and racial/ethnic differences across the nine US census divisions from 2001 to 2021 (excluding 2020 data) following the CDC guidance for data usage [8].

## 2. Materials and Methods

### 2.1. Study Design, Data Source, and Study Population

In this cross-sectional study, we analyzed data on HCC cases from the United States Cancer Statistics (USCS) database from 2001 to 2021, which covered 98% of the US population and included 50 states and the District of Columbia. USCS data are contributed by the National Cancer Institute’s (NCI) Surveillance, Epidemiology, and End Results (SEER) program, Centers for Disease Control and Prevention’s (CDC) National Program of Cancer Registries (NPCR), and CDC’s National Vital Statistics System (NVSS) [10]. All data analyzed in this study are de-identified and publicly available, and hence were exempted from review by the institutional review board based on the Policy for the National Human Research Protections Advisory Committee.

#### 2.1.1. Case Definition

HCC cases were identified according to the International Classification of Diseases for Oncology, Third Edition [ICD-O-3], site code C22.0 and histology codes 8170-8175, 8180/2, and 8180/3 [11].

#### 2.1.2. Study Variables

**Figure 1:**
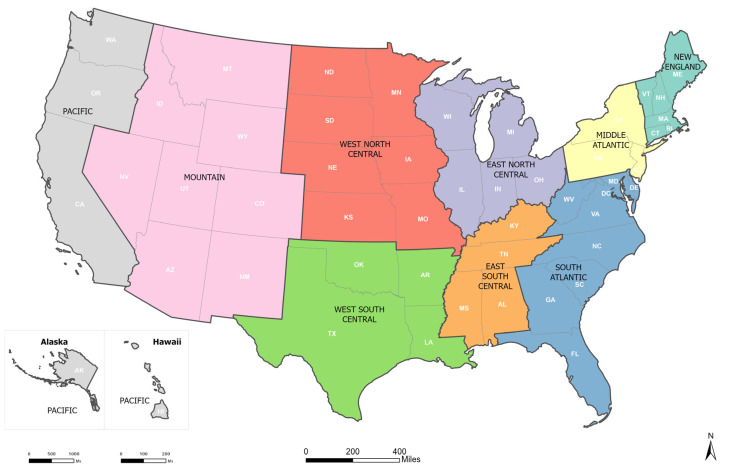
The nine US Census Bureau divisions. Footnote: US census divisions—Division 1: New England (Connecticut, Maine, Massachusetts, New Hampshire, Rhode Island, and Vermont), Division 2: Middle Atlantic (New Jersey, New York, and Pennsylvania), Division 3: East North Central (Illinois, Indiana, Michigan, Ohio, and Wisconsin), Division 4: West North Central (Iowa, Kansas, Minnesota, Missouri, Nebraska, North Dakota, and South Dakota), Division 5: South Atlantic (Delaware, Florida, Georgia, Maryland, North Carolina, South Carolina, Virginia, Washington D.C., and West Virginia), Division 6: East South Central (Alabama, Kentucky, Mississippi, and Tennessee), Division 7: West South Central (Arkansas, Louisiana, Oklahoma, and Texas), Division 8: Mountain (Arizona, Colorado, Idaho, Montana, Nevada, New Mexico, Utah, and Wyoming), and Division 9: Pacific (Alaska, California, Hawaii, Oregon, and Washington).

We assessed trends in age-adjusted incidence rates in the US overall, by census divisions, and then stratified by race/ethnicity. Geography was based on the nine U.S. Census Bureau divisions (Figure 1) [12,13]. Race/ethnicity was categorized as Hispanic (all races), non-Hispanic Asian or Pacific Islander, non-Hispanic Black, non-Hispanic White, and non-Hispanic American Indian/Alaska Native. We excluded other racial/ethnic subgroups due to small group sizes.

### 2.2. Analysis

The USCS database provides incidence rates per 100,000 population, age-adjusted to the 2000 US standard population. Rates were suppressed when the racial/ethnic group had fewer than 16 cases [14]. Joinpoint regression was performed to examine temporal trends in age-standardized incidence rates of HCC using the NCI’s Joinpoint Regression Program version 5.2.0. [15] Joinpoint regression fits a series of joined straight lines on a logarithmic scale to the trends in annual age-adjusted rates by starting with no joinpoint and then determining whether 1 or more joinpoints are required for the best model. If the annual percent change (APC) or average annual percentage change (AAPC) was statistically significantly different from zero, we considered the rates to increase or decrease; otherwise, we reported rates as stable or level. The statistical significance level was defined as a 2-sided *p*-value < 0.05. Of note, the Joinpoint program was recently updated with a feature that allows the exclusion of any data point. The change was made due to the effect of COVID-19 pandemic disruption on data collection, which may distort joinpoint modeling calculations. The new feature offers the opportunity to exclude potentially biased data points from joinpoint modeling calculations [8]. Thus, we conducted the joinpoint analysis using data from 2001 to 2021, excluding the 2020 dataset.

## 3. Results

### 3.1. HCC Incidence Rates, 2001–2021 (2020 Skipped)

The HCC age-adjusted incidence rates from 2001 to 2021 (excluding 2020 data) are presented in Table 1. There were 390,289 individuals diagnosed with HCC in the USCS registry during the study period. The overall national incidence rate was 5.49 per 100,000 population (95% confidence interval (CI): 5.47–5.50) during the study period. However, there were significant regional differences. The highest rate was in Division 7: West South Central (7.26 per 100,000 (95% CI: 7.20–7.32) and the lowest rate was observed in Division 4: West North Central (4.16 per 100,000 (95% CI: 4.10–4.22). For racial/ethnicity groups, the highest rate was found in the non-Hispanic Asian and Pacific Islander group, while the second and third highest rates were in the Hispanic and non-Hispanic American Indian/Alaska Native groups, respectively (Table 1).

### 3.2. Temporal Trends in HCC Incidence Rates

#### 3.2.1. Temporal Trends in HCC Incidence Rates Overall

**Figure 2:**
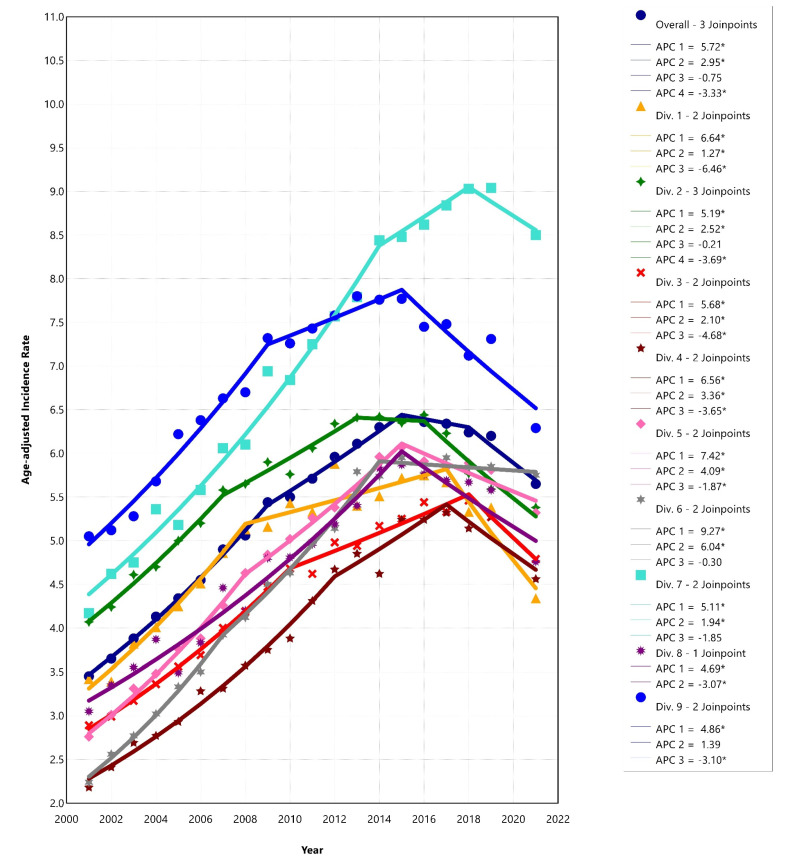
Trends in HCC age-adjusted incidence rates by US census geographic divisions, 2001–2021. Footnotes: *y* axis: age-standardized incidence rate per 100,000. * Indicates that the Annual Percent Change (APC) is significantly different from zero at the alpha = 0.05 level. Abbreviations: APC, annual percent change; Div, US census division. Trend analyses did not include 2020 data because the Centers for Disease Control and Prevention (CDC), in their cautionary recommendations [8], stated that due to cancer data reporting disruptions during the COVID-19 pandemic, using 2020 data may distort joinpoint trend analysis results. APC 1, 2, and 3 represent temporal joinpoint segments based on annual percent changes in age-adjusted HCC incidence rates. US census divisions—Division 1: New England (Connecticut, Maine, Massachusetts, New Hampshire, Rhode Island, and Vermont), Division 2: Middle Atlantic (New Jersey, New York, and Pennsylvania), Division 3: East North Central (Illinois, Indiana, Michigan, Ohio, and Wisconsin), Division 4: West North Central (Iowa, Kansas, Minnesota, Missouri, Nebraska, North Dakota, and South Dakota), Division 5: South Atlantic (Delaware, Florida, Georgia, Maryland, North Carolina, South Carolina, Virginia, Washington D.C., and West Virginia), Division 6: East South Central (Alabama, Kentucky, Mississippi, and Tennessee), Division 7: West South Central (Arkansas, Louisiana, Oklahoma, and Texas), Division 8: Mountain (Arizona, Colorado, Idaho, Montana, Nevada, New Mexico, Utah, and Wyoming), and Division 9: Pacific (Alaska, California, Hawaii, Oregon, and Washington).

The AAPC and APC of the joinpoint regression analyses are displayed in Table 2 and Supplementary Table S1. There was an increase in HCC incidence rates overall (AAPC = 2.51, 95% CI: 2.32–2.72, *p* < 0.001) from 2001 to 2021. However, the joinpoint analysis for the national trend identified sub trends within the overall time period; it showed that HCC incidence rates increased from 2001 until 2015, stabilized during 2015–2018 (APC = −0.75, 95% CI: −1.31–3.26, *p* = 0.453), and then decreased by 3% per year from 2018 to 2021 (APC = −3.33, 95% CI: −4.78–−1.96, *p* < 0.001) (Figure 2).

#### 3.2.2. Temporal Trends in HCC Incidence in US Census Divisions

HCC incidence rates increased across each US census division during the entire study period. However, most divisions have experienced a downward trend in HCC incidence in recent years, except Divisions 6: East South Central (2014–2021: APC = −0.30, 95% CI: −1.08–0.37, *p* = 0.294) and Division 7: West South Central (2018–2021: APC = −1.85, 95% CI: −3.52–0.52, *p* = 0.136), where trends leveled (Figure 2). The steepest decreases were observed in Division 1: New England (2017–2021: APC = −6.46, 95% CI: −9.62–−3.96, *p* < 0.001) and Division 3: East North Central (2018–2021: APC = −4.68, 95% CI: −6.27–−2.78, *p* < 0.001).

#### 3.2.3. Temporal Trends in HCC Incidence in US Census Divisions by Race and Ethnicity

**Figure 3:**
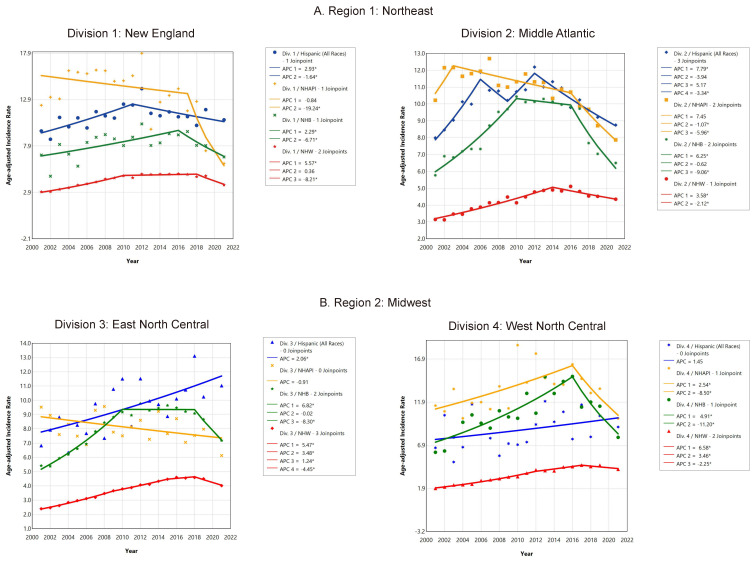

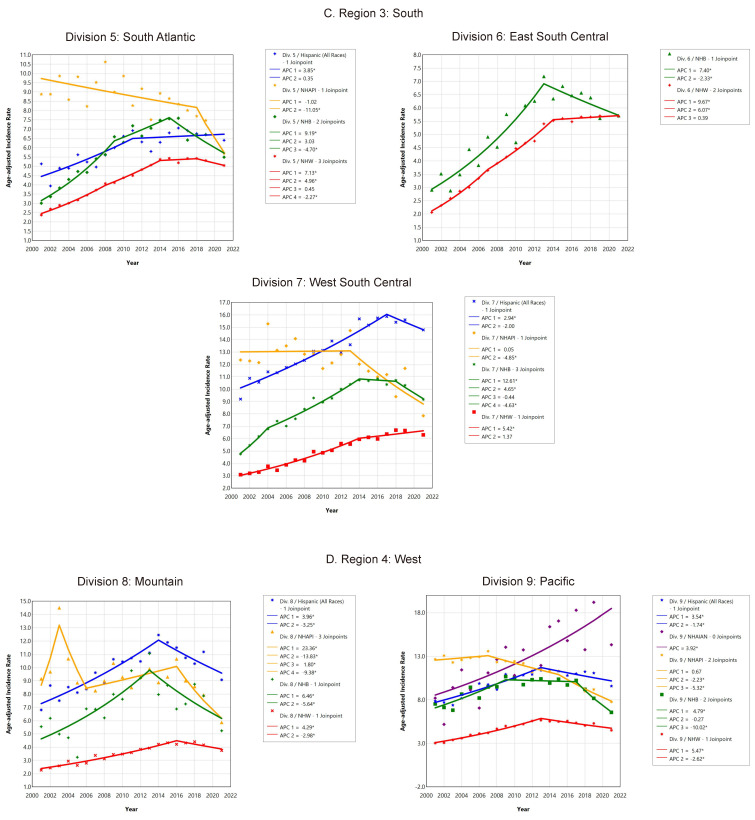
(**A**–**D**) Trends in HCC age-adjusted incidence rates by US census divisions and by race/ethnicity, 2001–2021. Footnotes: *y* axis: age-standardized incidence rate per 100,000. * Indicates that the Annual Percent Change (APC) is significantly different from zero at the alpha = 0.05 level. Abbreviations: APC, annual percent change; Div, US census division. Trend analyses did not include 2020 data because the Centers for Disease Control and Prevention (CDC), in their cautionary recommendations [8], stated that due to cancer data reporting disruptions during the COVID-19 pandemic, using 2020 data may distort joinpoint trend analysis results. APC 1, 2, and 3 represent temporal joinpoint segments based on annual percent changes in age-adjusted HCC incidence rates. US census divisions—Division 1: New England (Connecticut, Maine, Massachusetts, New Hampshire, Rhode Island, and Vermont), Division 2: Middle Atlantic (New Jersey, New York, and Pennsylvania), Division 3: East North Central (Illinois, Indiana, Michigan, Ohio, and Wisconsin), Division 4: West North Central (Iowa, Kansas, Minnesota, Missouri, Nebraska, North Dakota, and South Dakota), Division 5: South Atlantic (Delaware, Florida, Georgia, Maryland, North Carolina, South Carolina, Virginia, Washington D.C., and West Virginia), Division 6: East South Central (Alabama, Kentucky, Mississippi, and Tennessee), Division 7: West South Central (Arkansas, Louisiana, Oklahoma, and Texas), Division 8: Mountain (Arizona, Colorado, Idaho, Montana, Nevada, New Mexico, Utah, and Wyoming), and Division 9: Pacific (Alaska, California, Hawaii, Oregon, and Washington).

Joinpoint analyses of census divisions stratified by race and ethnicity are presented in Figure 3A–D and Supplementary Table S1. We found substantial racial/ethnic variations in HCC incidence across US census divisions. For Hispanics, HCC incidence increased in most divisions over the duration of the study, except in Division 1: New England (AAPC = 0.62, 95% CI: −0.34–2.08, *p* = 0.134) and Division 4: West North Central (AAPC = 1.45, 95% CI: −0.62–4.31, *p* = 0.149), where the increases were not significant. Temporal changes could not be assessed for non-Hispanic American Indian/Alaska Native individuals in eight of the nine divisions due to small sample sizes. However, in the only available trend (Division 9: Pacific), there was a substantial upsurge in incidence rates throughout the study period for American Indian/Alaska Native individuals (AAPC = 3.92, 95% CI: 2.03–6.60, *p* < 0.001). For the Non-Hispanic Asian and Pacific Islander group, HCC incidence decreased in many divisions (Divisions 1, 2, 5, 7, 8, and 9) but stabilized in other divisions (Divisions 3, and 4) over time. A joinpoint could not be determined for the group in Division 6: East South Central due to the suppressed small sample size. Among non-Hispanic Black individuals, HCC incidence either increased or leveled across US divisions during the entire study period. For example, rates increased in Divisions 3, 5, 6, and 7 during the entire study period. For non-Hispanic White individuals, HCC incidence rose from 2001 to 2021 across every division, with the largest rise in Division 6: East South Central (AAPC = 5.09, 95% CI: 4.88–5.38, *p* < 0.001).

#### 3.2.4. Recent Temporal Trends in HCC Incidence in US Census Divisions by Race and Ethnicity

In recent years, joinpoint analyses (Figure 3A–D) indicated downward trends in HCC incidence among multiple racial/ethnic groups across the US census divisions. Among Hispanic populations, there was downward trend in incidence rates in multiple census divisions (1, 2, 8, and 9), while they stabilized in Divisions 4, 5, and 7 and increased in Division 3. For the non-Hispanic American Indian/Alaska Native group in Division 9: Pacific, there was no inflection point, and the rate increased (APC = 3.92, 95% CI: 2.03–6.60, *p* < 0.001). Among Non-Hispanic Asian and Pacific Islanders, sharp decreases were observed in all divisions except Division 3: East North Central, where rates plateaued (2001–2021: APC = −0.91, 95% CI: −1.99–0.44, *p* = 0.184) throughout the study period. The greatest rate declines among Non-Hispanic Asian and Pacific Islander populations were seen in Division 1: New England from 2017 to 2021 (APC = −19.24, 95% CI: −32.22–−10.08, *p* < 0.001). Joinpoint analyses suggested significant slowing in HCC incidence trends in recent years among Non-Hispanic Black populations in every division. We found the highest recent rate declines among Non-Hispanic Black people in Division 4: West North Central from 2016 to 2021 (APC = −11.20, 95% CI: −24.97–−4.43, *p* = 0.006), followed by Division 2: Middle Atlantic (APC = −9.06, 95% CI: −12.45–−6.90, *p* < 0.001). Lastly, for the non-Hispanic White group, a substantial downward trend in HCC incidence has occurred in recent years in seven of the nine divisions (starting from 2018 in some of the divisions), but this plateaued in Divisions 6 and 7.

## 4. Discussion

This temporal trend analysis of HCC incidence rates in the United States found a national increasing trend from 2001 to 2021. However, the trend was not linear. While rates increased rapidly through 2009, the rate of increase has slowed, plateaued, or even reversed (decreased) in recent years. In addition, we found variations across subgroups of the population when the trend analysis was stratified by US census divisions and race/ethnicity. Of note, southern US divisions had higher HCC incidence rates from 2001 to 2021, with Division 6 (East South Central) having the highest rate and AAPC. While the current analysis found decreased rates in most divisions in recent years, rates in the southern divisions remained stable over the period. Furthermore, in recent years, incidence trends have decreased considerably for the non-Hispanic Black and non-Hispanic Asian and Pacific Islander groups in almost all divisions, while they varied (either stabilizing, increasing, or decreasing) for non-Hispanic White and Hispanic populations across US census divisions. Also, in recent years, the rate has increased significantly among the non-Hispanic American Indian/Alaska Native group.

Our national data showing a non-linear upward but slowing trend in HCC incidence over the study period are in contrast with recent studies of non-national (SEER) datasets suggesting a declining or stable trend [3,4,5]. While the reasons for slowing rates of increase, as well as a downtrend in recent years, may be not be completely understood, the changes may be partly due to ongoing HCC interventions, including viral hepatitis prevention (e.g., vaccination (HBV) and screening (HBV and HCV)) [16], antiviral treatment for HCV and HBV, and tobacco cessation programs [17] and treatments [18]. A possible explanation for the observed geographic and racial/ethnic variations in HCC trends may be due to varying levels of exposure to risk factors [19,20,21,22], population characteristics, and inequitable access to prevention or guideline-concordant treatments [23,24]. The shifting landscape of HCC risk factors [25], which impact subgroups differently, may contribute to racial/ethnic and place-based HCC trend variations in the current study. Non-Hispanic Asian or Pacific Islanders were 10 times more at risk for chronic HBV, and non-Hispanic American Indian and Alaskan Natives had twice the risk of HCV than non-Hispanic White individuals [26,27,28]. However, the trend of HBV- and HCV-related HCC incidence and mortality have drastically reduced over the years due to effective preventative measures [26,27,28]. Interventions targeting specific racial/ethnic groups with high burdens of HCC risk factors may have contributed to the observed trend [29].

Notable favorable trends among the non-Hispanic Asian and Pacific Islanders and non-Hispanic Black groups may be due to increased awareness, improved screening uptake, vaccination (HBV), and treatments (HCV and HBV). Despite reducing infection-related (HBV and HCV) HCC incidence, the rapidly rising prevalence of MASLD [30] and alcohol-associated liver diseases are possible drivers of the observed national increasing HCC incidence trends. Moreover, studies have reported increasing mortality from MASLD and alcohol-related liver diseases in the US [31,32,33,34]. Metabolic dysfunctions, e.g., diabetes, obesity, and MASLD, are more prevalent among Hispanic populations compared to other ethnic subgroups [21]. The conditions are challenging to prevent and treat [35], and possibly slow down the rates of HCC incidence decline in this group. The American Indian/Alaskan Native group experiences a disproportionately higher prevalence of alcohol use disorder and MASLD than any other racial group in the US [36,37]. Unfavorable HCC incidence trends in the group may be partly related to the exacerbation of these risks by multilevel stressors, including individual and area-level low SES and structural discrimination, coupled with limited access to coping resources.

Place-based determinants of health across racial/ethnic groups influence cancer risks and incidence. Geographic variation in HCC incidence in the US is well documented [28,38].Population characteristics and healthcare access differences could partly explain US census division disparities in HCC trends. Despite decreasing HCC incidence trends in recent years in most US divisions, rates remained stable in the East South Central (Alabama, Kentucky, Mississippi, and Tennessee) and West South Central (Arkansas, Louisiana, Oklahoma, and Texas) US. Historically, the southern US, in general, has had higher-than-national proportions of Hispanics and low socioeconomic status (SES). The geographic disparities in HCC incidence trends likely represent a constellation of HCC risk factor burden and poor access to preventative services in these divisions [39,40,41,42]. Our data highlight areas of opportunity for targeted HCC prevention. Studies are needed to understand the multilevel determinants contributing to slow progress in HCC prevention, including biological, behavioral, social, environmental, and health system factors. Optimizing risk stratification, availability and access to effective HCC interventions, and pharmacological management, as well as prioritizing inclusiveness, are essential to reducing disparities in HCC incidence. There is a need to develop targeted evidence-based intervention studies within clinical and community settings to increase awareness about HCC risk and prevention, and behaviors to curb emerging modifiable risks, including obesity, MASLD, and alcohol use disorder.

### Limitations

This study has important limitations that are worth considering when interpreting our results. First, the USCS data do not provide information on risk factors. Thus, we could not examine the role of specific exposures that may influence HCC incidence. Second, the results of some groups were suppressed due to small sample sizes to minimize the risk of patient confidentiality disclosure. Third, we cannot rule out the possibility of misclassification of race and ethnicity during data collection. Fourth, data for racial/ethnic groups with small sizes (e.g., non-Hispanic American Indian/Alaskan Native) were suppressed for confidentiality reasons, so the interpretation of the groups’ results should be approached with caution due to the possibility of errors arising from missing data. However, the strengths of our study are the use of representative data covering all 50 US states and the District of Columbia, as well as a comprehensive analysis examining trends in smaller geographic units for multiple racial and ethnic groups, unlike recent studies conducted at the national level. For example, our study is the first to report a significant downward trend in HCC incidence among non-Hispanic Black populations in every US census division. Also, while most recent studies have reported declining rates among non-Hispanic Asian and Pacific Islanders in general, we found stabilized rates for those in Division 3 (East North Central).

## 5. Conclusions

Our data showed a slowing uptrend in HCC incidence nationally. Southern US census divisions had higher incidence rates and a more rapid uptick than other divisions from 2001 to 2021, with the highest rate in Division 6 (East South Central). Unlike other divisions with downward trends over time, trends only leveled in Division 6 (East South Centra) and Division 7 (West South Central). Across US census divisions, the greatest declines in rates were observed among non-Hispanic Asian and Pacific Islanders, followed by non-Hispanic Black. The Non-Hispanic White and Hispanic groups were the least affected by the recent downward trend, while non-Hispanic American Indians/Alaska Natives exhibited an upward trend. Targeted interventions are needed to reduce HCC disparities in the US.

## Figures and Tables

**Table 1:** HCC age-adjusted incidence rates for US population by census divisions, sex, and race/ethnicity, 2001–2021.

Population	Age-Adjusted Incidence Rate (95% CI)	Count (*n*)
National (50 States and District of Columbia)	5.49 (5.47 to 5.50)	390,289
US Census Divisions		
Division 1: New England	5.02 (4.95–5.1)	17,923
Division 2: Middle Atlantic	5.69 (5.64–5.74)	56,391
Division 3: East North Central	4.55 (4.51–4.59)	49,227
Division 4: West North Central	4.16 (4.1–4.22)	20,045
Division 5: South Atlantic	5 (4.96–5.03)	72,464
Division 6: East South Central	4.79 (4.72–4.86)	20,611
Division 7: West South Central	7.26 (7.2–7.32)	55,060
Division 8: Mountain	4.91 (4.84–4.97)	23,825
Division 9: Pacific	6.95 (6.9–7.0)	74,743
Race and Ethnicity		
Hispanic (All Races)	10.21 (10.12–10.29)	63,039
Non-Hispanic American Indian/Alaska Native	9.46 (9.17–9.76)	4326
Non-Hispanic Asian or Pacific Islander	10.66 (10.54–10.78)	32,203
Non-Hispanic Black	7.68 (7.61–7.74)	57,874
Non-Hispanic White	4.29 (4.27–4.31)	231,319
Non-Hispanic Unknown Race	~	1528
Sex		
Female	2.40 (2.38 to 2.42)	90,797
Male	9.03 (9.00 to 9.06)	299,492
Age		
<50 years	0.66 (0.65–0.67)	27,443
50+ years	18.13 (18.07–18.19)	362,846

**Table 2:** Trends in HCC incidence rates among US population overall and by US census divisions, 2001 to 2021.

Population	Joinpoint Segment		APC (95% CI)	*p*	Year 2001–2021	*p*
	Year Start	Year End			AAPC (95% CI)	
Overall						
	2001	2009	5.72 (4.56–6.59)	<0.001	2.51 (2.32–2.72)	<0.001
	2009	2015	2.95 (2.39–6.77)	<0.001		
	2015	2018	−0.75 (−1.31–3.26)	0.453		
	2018	2021	−3.33 (−4.78–−1.96)	<0.001		
Division 1: New England (Connecticut, Maine, Massachusetts, New Hampshire, Rhode Island, Vermont)						
	2001	2008	6.64 (5.17–9.39)	<0.001	1.50 (1.02–2.00)	<0.001
	2008	2017	1.27 (0.16–2.36)	0.034		
	2017	2021	−6.46 (−9.62–−3.96)	<0.001		
Division 2: Middle Atlantic (New Jersey, New York, Pennsylvania)						
	2001	2007	5.19 (2.87–8.06)	<0.001	1.30 (1.04–1.58)	<0.001
	2007	2013	2.52 (1.48–6.95)	<0.001		
	2013	2016	−0.21 (−2.54–2.75)	0.961		
	2016	2021	−3.69 (−5.36–−2.75)	<0.001		
Division 3: East North Central (Illinois, Indiana, Michigan, Ohio, Wisconsin)						
	2001	2010	5.68 (5.20–6.31)	<0.001	2.63 (2.40–2.85)	<0.001
	2010	2018	2.10 (1.65–2.59)	<0.001		
	2018	2021	−4.68 (−6.27–−2.78)	<0.001		
Division 4: West North Central (Iowa, Kansas, Minnesota, Missouri, Nebraska, North Dakota, South Dakota)						
	2001	2012	6.56 (5.91–9.74)	0.001	3.64 (3.19–4.20)	<0.001
	2012	2017	3.36 (1.12–5.69)	0.008		
	2017	2021	−3.65 (−7.00–−1.54)	0.005		
Division 5: South Atlantic (Delaware, Florida, Georgia, Maryland, North Carolina, South Carolina, Virginia, Washington D.C., West Virginia)						
	2001	2008	7.42 (6.62–8.72)	<0.001	3.40 (3.18–3.67)	<0.001
	2008	2015	4.09 (3.10–4.74)	<0.001		
	2015	2021	−1.87 (−2.69–−1.11)	<0.001		
Division 6: East South Central (Alabama, Kentucky, Mississippi, Tennessee)						
	2001	2007	9.27 (8.01–11.94)	<0.001	4.71 (4.47–5.06)	<0.001
	2007	2014	6.04 (4.65–6.86)	0.002		
	2014	2021	−0.30 (−1.08–0.37)	0.294		
Division 7: West South Central (Arkansas, Louisiana, Oklahoma, Texas)						
	2001	2014	5.11 (3.48–6.78)	0.004	3.40 (3.14–3.76)	<0.001
	2014	2018	1.94 (0.77–5.93)	0.003		
	2018	2021	−1.85 (−3.52–0.52)	0.136		
Division 8: Mountain (Arizona, Colorado, Idaho, Montana, Nevada, New Mexico, Utah, Wyoming)						
	2001	2015	4.69 (4.04–5.54)	<0.001	2.30 (1.80–2.83)	<0.001
	2015	2021	−3.07 (−5.74–−1.32)	0.002		
Division 9: Pacific (Alaska, California, Hawaii, Oregon, Washington)						
	2001	2009	4.86 (4.05–6.77)	0.001	1.38 (1.05–1.73)	<0.001
	2009	2015	1.39 (−0.42–2.93)	0.096		
	2015	2021	−3.10 (−4.96–−2.07)	<0.001		

Abbreviations: HCC, hepatocellular carcinoma; APC, annual percentage change; and AAPC, average annual percentage change. Trend analyses did not include 2020 data because the Centers for Disease Control and Prevention (CDC), in their cautionary recommendations [8], stated that due to cancer data reporting disruptions during the COVID-19 pandemic, using 2020 data may distort joinpoint trend analysis results.

## Data Availability

The data used in the current study are de-identified and publicly available and can be requested and obtained from the USCS website.

## Supplementary Materials

The following supporting information can be downloaded at https://www.mdpi.com/article/10.3390/cancers17091431/s1: Table S1: Trends in HCC incidence rates among US Population by US Census Divisions and Race/Ethnicity, 2001 to 2021 (2020 skipped).

## References

[1] National Cancer Institute (NCI) Liver Cancer Causes, Risk Factors, and Prevention. https://www.cancer.gov/types/liver/what-is-liver-cancer/causes-risk-factors.

[2] American Cancer Society Key Statistics About Liver Cancer. https://www.cancer.org/cancer/types/liver-cancer/about/what-is-key-statistics.html.

[3] Thrift A.P., Liu K.S., Raza S.A., El-Serag H.B. (2023). Recent Decline in the Incidence of Hepatocellular Carcinoma in the United States. Clin. Gastroenterol. Hepatol..

[4] O’Brien T.R., Devesa S.S., Koshiol J., Marrero J.A., Shiels M.S. (2023). Decreasing incidence of hepatocellular carcinoma among most racial groups: SEER-22, 2000–2019. Cancer Med..

[5] Kaur B., Yeo Y.H., Liang J., Luu M., Ayoub W., Kuo A., Trivedi H., Sankar K., Gong J., Hendifar A. (2023). COVID-19 Pandemic Impact on Diagnosis, Stage, and Treatment of Hepatocellular Carcinoma in the United States. Gastro. Hep. Adv..

[6] Abboud Y., Ismail M., Khan H., Medina-Morales E., Alsakarneh S., Jaber F., Pyrsopoulos N.T. (2024). Hepatocellular Carcinoma Incidence and Mortality in the USA by Sex, Age, and Race: A Nationwide Analysis of Two Decades. J. Clin. Transl. Hepatol..

[7] United States Census Bureau Statistical Groupings of States and Counties. https://www2.census.gov/geo/pdfs/reference/GARM/Ch6GARM.pdf.

[8] U.S. Cancer Statistics Cautionary Notes. https://www.cdc.gov/united-states-cancer-statistics/public-use/cautionary-notes.html#cdc_generic_section_2-impact-of-covid-19-on-cancer-incidence-data.

[9] National Cancer Institute, Division of Cancer Control&Population Sciences Joinpoint Revision History. https://surveillance.cancer.gov/help/joinpoint/tech-help/joinpoint-revision-history.

[10] Center for Disease Control and Prevention. U.S. Cancer Statistics. https://www.cdc.gov/united-states-cancer-statistics/technical-notes/index.html.

[11] National Cancer Institute, SEER Training Modules ICD-O-3 Site Codes. https://training.seer.cancer.gov/biliary/abstract-code-stage/codes.html.

[12] U.S. Census Bureau (2020). United States Census History: Regions and Divisions. US Census Bureau Website. https://www.census.gov/history/www/programs/geography/regions_and_divisions.html.

[13] U.S. Census Bureau (2019). Census Regions and Divisions of the United States.

[14] U.S. Cancer Statistics. Cautionary Notes. https://www.cdc.gov/united-states-cancer-statistics/public-use/cautionary-notes.html#:~:text=The%20decline%20in%20cancer%20incidence,one%2Dyear%20anomaly%20in%20data.

[15] (2024). Joinpoint Regression Program.

[16] Zhang X., Guan L., Tian H., Zeng Z., Chen J., Huang D., Sun J., Guo J., Cui H., Li Y. (2021). Risk Factors and Prevention of Viral Hepatitis-Related Hepatocellular Carcinoma. Front. Oncol..

[17] American Cancer Society Health Benefits of Quitting Smoking Over Time. https://www.cancer.org/cancer/risk-prevention/tobacco/benefits-of-quitting-smoking-over-time.html.

[18] Mak L.-Y., Cruz-Ramón V., Chinchilla-López P., Torres H.A., LoConte N.K., Rice J.P., Foxhall L.E., Sturgis E.M., Merrill J.K., Bailey H.H. (2018). Global Epidemiology, Prevention, and Management of Hepatocellular Carcinoma. Am. Soc. Clin. Oncol. Educ. Book.

[19] U.S. Centers for Disease Control and Prevention Hepatitis C. https://www.cdc.gov/hepatitis/statistics/2022surveillance/hepatitis-c.htm#:~:text=During%202022%2C%20rates%20of%20acute,common%20was%20injection%20drug%20use.

[20] Kim H.S., Rotundo L., Yang J.D., Kim D., Kothari N., Feurdean M., Ruhl C., Unalp-Arida A. (2017). Racial/ethnic disparities in the prevalence and awareness of Hepatitis B virus infection and immunity in the United States. J. Viral. Hepat..

[21] Tesfai K., Pace J., El-Newihi N., Martinez M.E., Tincopa M.A., Loomba R. (2024). Disparities for Hispanic Adults With Metabolic Dysfunction-associated Steatotic Liver Disease in the United States: A Systematic Review and Meta-analysis. Clin. Gastroenterol. Hepatol..

[22] Gurka M.J., Filipp S.L., DeBoer M.D. (2018). Geographical variation in the prevalence of obesity, metabolic syndrome, and diabetes among US adults. Nutr. Diabetes.

[23] Castaneda D., Gonzalez A.J., Alomari M., Tandon K., Zervos X.B. (2021). From hepatitis A to E: A critical review of viral hepatitis. World J. Gastroenterol..

[24] U.S. Department of Health and Human Services Office of Minority Health Hepatitis and African Americans. https://minorityhealth.hhs.gov/hepatitis-and-african-americans.

[25] Sagnelli E., Macera M., Russo A., Coppola N., Sagnelli C. (2020). Epidemiological and etiological variations in hepatocellular carcinoma. Infection.

[26] Bradley H., Hall E.W., Rosenthal E.M., Sullivan P.S., Ryerson A.B., Rosenberg E.S. (2020). Hepatitis C Virus Prevalence in 50 U.S. States and D.C. by Sex, Birth Cohort, and Race: 2013–2016. Hepatol. Commun..

[27] Mera J., Joshi K., Thornton K., Box T., Scott J., Sedillo M., Deming P., David C., Essex W., Manch R. (2019). Retrospective Study Demonstrating High Rates of Sustained Virologic Response After Treatment With Direct-Acting Antivirals Among American Indian/Alaskan Natives. Open Forum. Infect. Dis..

[28] Herren O.M., Gillman A.S., Marshall V.J., Das R. (2023). Understanding the Changing Landscape of Health Disparities in Chronic Liver Diseases and Liver Cancer. Gastro Hep Adv..

[29] The Hepatitis B Foundation Hepatitis B in Asian Populations. https://www.hepb.org/blog/hepatitis-b-asian-populations/.

[30] Chan W.K., Chuah K.H., Rajaram R.B., Lim L.L., Ratnasingam J., Vethakkan S.R. (2023). Metabolic Dysfunction-Associated Steatotic Liver Disease (MASLD): A State-of-the-Art Review. J. Obes. Metab. Syndr..

[31] Sun M., Sun H. (2025). Recent prevalence and trends of obesity and metabolic dysfunction-associated steatotic liver disease (MASLD) among US adolescents: 1999 to 2020. Pediatr. Obes..

[32] Termeie O., Fiedler L., Martinez L., Foster J., Perumareddi P., Levine R.S., Hennekens C.H. (2022). Alarming trends: Mortality from alcoholic cirrhosis in the United States. Am. J. Med..

[33] Yoon Y.H., Chen C.M., Slater M.E., Jung M.K., White A.M. (2020). Trends in Premature Deaths From Alcoholic Liver Disease in the U.S., 1999–2018. Am. J. Prev. Med..

[34] Ilyas F., Ali H., Patel P., Basuli D., Giammarino A., Satapathy S.K. (2023). Rising alcohol-associated liver disease-related mortality rates in the United States from 1999 to 2022. Hepatol. Commun..

[35] U.S. National Heart, Lung, and Blood Institute Metabolic Syndrome: Treatment. https://www.nhlbi.nih.gov/health/metabolic-syndrome/treatment#:~:text=Choose%20heart%2Dhealthy%20foods,Get%20enough%20good%20quality%20sleep.

[36] Emerson M.A., Moore R.S., Caetano R. (2017). Association Between Lifetime Posttraumatic Stress Disorder and Past Year Alcohol Use Disorder Among American Indians/Alaska Natives and Non-Hispanic Whites. Alcohol. Clin. Exp. Res..

[37] Aboona M.B., Faulkner C., Rangan P., Ng C.H., Huang D.Q., Muthiah M., Nevah Rubin M.I., Han M.A.T., Fallon M.B., Kim D. (2024). Disparities among ethnic groups in mortality and outcomes among adults with MASLD: A multicenter study. Liver Int..

[38] El-Serag H.B., Kanwal F. (2014). Epidemiology of hepatocellular carcinoma in the United States: Where are we? Where do we go?. Hepatology.

[39] KFF Status of State Medicaid Expansion Decisions: Interactive Map. https://www.kff.org/affordable-care-act/issue-brief/status-of-state-medicaid-expansion-decisions-interactive-map/#:~:text=Coverage%20under%20the%20Medicaid%20expansion,%2C%20Virginia%20(1%2F1%2F.

[40] U.S. Census Bureau Quick Facts United States. https://www.census.gov/quickfacts/US.

[41] Kaiser State Health Facts (2021). Health Insurance Coverage in the United States. Uninsured Rates for the Nonelderly by Age. https://www.kff.org/state-category/health-coverage-uninsured/.

[42] Montero A., Kearney A., Hamel L., Lopes L. Americans’ Challenges with Health Care Costs. https://www.kff.org/health-costs/issue-brief/americans-challenges-with-health-care-costs/.

