# Supplementary material for: Differences in Hepatocellular Carcinoma Incidence Trends Across US Census Divisions, 2001 to 2021
Source: Cancers (Basel). 2025 Apr 24;17(9):1431. doi: 10.3390/cancers17091431 (PMC12070962; doi:10.3390/cancers17091431)
Supplement: Supplementary file 1 [file cancers-17-01431-s001.zip › cancers-3533781-supplementary.pdf]

Differences in Hepatocellular Carcinoma Incidence Trends Across US Census Divisions, 2001 to 2021

Itunu O. Sokale [1,2,*], Omar Rosales [1], Aaron P. Thrift [1,2], Hashem B. El-Serag [2,3], Elyse Burgess [1] and Abiodun O. Oluyomi [1,2,4]

1. Section of Epidemiology and Population Sciences, Department of Medicine, Baylor College of Medicine, Houston, TX 77030, USA; oluyomi@bcm.edu (A.O.O.)
2. Dan L. Duncan Comprehensive Cancer Center, Baylor College of Medicine, Houston, TX 77030, USA
3. Section of Gastroenterology and Hepatology and Clinical Epidemiology and Comparative Effectiveness Program in the Health Services Research, Michael E. DeBakey VA Medical Center, Baylor College of Medicine, Houston, TX 77030, USA
4. Center for Precision Environmental Health, Baylor College of Medicine, Houston, TX 77030, USA

* Correspondence: itunu.sokale@bcm.edu

Supplementary Table S1. Trends in HCC incidence rates among US Population by US Census Divisions and Race/Ethnicity, 2001 to 2021 (2020 skipped)

Population	Joinpoint Segment Year Start	Joinpoint Segment Year End	APC (95% CI)	*P*	Year 2001-2021 AAPC (95% CI)	*P*	Parallel Pairwise Comparison P-Value[a,b]
**Division 1:** New England (Connecticut, Maine, Massachusetts, New Hampshire, Rhode Island, Vermont)							
Hispanic (All Races)	2001	2011	2.93 (1.20-11.09)	0.002	0.62 (-0.34-2.08)	0.134	< 0.001
	2011	2021	-1.64 (-5.63--0.11)	0.036			
Non-Hispanic American Indian/Alaska Native	-	-	-	-	-	-	-
Non-Hispanic Asian or Pacific Islander	2001	2017	-0.84 (-2.22-1.36)	0.496	-4.82 (-6.73--3.11)	0.002	< 0.001
	2017	2021	-19.24 (-32.22--10.08)	< 0.001			
Non-Hispanic Black	2001	2016	2.29 (1.07-6.53)	0.001	-0.04 (-1.55-1.87)	0.898	0.134
	2016	2021	-6.71 (-17.60--0.81)	0.022			
Non-Hispanic White	2001	2010	5.57 (4.79-6.65)	< 0.001	1.31 (0.91-1.67)	< 0.001	
	2010	2018	0.36 (-0.43-1.25)	0.286			
	2018	2021	-8.21 (-10.60--5.35)	< 0.001			
**Division 2:** Middle Atlantic (New Jersey, New York, Pennsylvania)							
Hispanic (All Races)	2001	2006	7.79 (5.98-11.77)	0.001	0.5 (0.12-1.02)	0.012	< 0.001
	2006	2009	-3.94 (-6.46-0.88)	0.084			
	2009	2012	5.17 (-2.28-7.76)	0.063			
	2012	2021	-3.34 (-4.46--2.55)	0.002			
Non-Hispanic American Indian/Alaska Native	-	-	-	-	-	-	-
Non-Hispanic Asian or Pacific Islander	2001	2003	7.45 (-0.58-15.61)	0.083	-1.51 (-2.21--0.78)	< 0.001	< 0.001
	2003	2016	-1.07 (-2.14--0.20)	0.045			
	2016	2021	-5.96 (-9.61--4.10)	< 0.001			
Non-Hispanic Black	2001	2010	6.25 (5.14-8.09)	< 0.001	0.17 (-0.37-0.71)	0.519	0.005
	2010	2016	-0.62 (-2.97-1.73)	0.526			
	2016	2021	-9.06 (-12.45--6.90)	< 0.001			
Non-Hispanic White	2001	2014	3.58 (3.07-4.31)	< 0.001	1.55 (1.15-1.94)	< 0.001	
	2014	2021	-2.12 (-3.84--0.86)	0.001			
**Division 3:** East North Central (Illinois, Indiana, Michigan, Ohio, Wisconsin)							
Hispanic (All Races)	2001	2021	2.06 (0.80-3.80)	0.004	2.06 (0.80-3.80)	0.004	0.032
Non-Hispanic American Indian/Alaska Native	-	-	-	-	-	-	-
Non-Hispanic Asian or Pacific Islander	2001	2021	-0.91 (-1.99-0.44)	0.184	-0.91 (-1.99-0.44)	0.184	< 0.001
Non-Hispanic Black	2001	2010	6.82 (5.95-8.06)	< 0.001	1.68 (1.22-2.09)	< 0.001	
	2010	2018	-0.02 (-0.75-0.92)	0.945			
	2018	2021	-8.30 (-11.42--5.43)	< 0.001			
Non-Hispanic White	2001	2009	5.47 (4.35-7.14)	< 0.001	2.69 (2.48-2.92)	< 0.001	< 0.001
	2009	2015	3.48 (2.96-6.44)	< 0.001			
	2015	2018	1.24 (0.49-3.25)	0.005			
	2018	2021	-4.45 (-6.10--2.99)	< 0.001			

Supplementary Table S1 (continued). Trends in HCC incidence rates among US Population by US Census Divisions and Race/Ethnicity, 2001 to 2021 (2020 skipped)

Population	Joinpoint Segment Year Start	Joinpoint Segment Year End	APC (95% CI)	*P*	Year 2001-2021 AAPC (95% CI)	*P*	Parallel Pairwise Comparison P-Value[a,b]
**Division 4:** West North Central (Iowa, Kansas, Minnesota, Missouri, Nebraska, North Dakota, South Dakota)							
Hispanic (All Races)	2001	2021	1.45 (-0.62-4.31)	0.149	1.45 (-0.62-4.31)	0.149	0.007
Non-Hispanic American Indian/Alaska Native	-	-	-	-	-	-	-
Non-Hispanic Asian or Pacific Islander	2001	2016	2.54 (1.13-5.69)	0.002	-0.34 (-1.56-1.31)	0.697	< 0.001
	2016	2021	-8.50 (-17.04--2.40)	0.003			
Non-Hispanic Black	2001	2016	4.91 (3.36-7.87)	< 0.001	0.63 (-1.71-2.52)	0.31	0.005
	2016	2021	-11.20 (-24.97--4.43)	0.006			
Non-Hispanic White	2001	2012	6.58 (5.74-9.88)	0.002	3.98 (3.56-4.51)	< 0.001	
	2012	2017	3.46 (1.52-6.38)	0.002			
	2017	2021	-2.25 (-5.23--0.01)	0.049			
**Division 5:** South Atlantic (Delaware, Florida, Georgia, Maryland, North Carolina, South Carolina, Virginia, Washington D.C., West Virginia)							
Hispanic (All Races)	2001	2011	3.85 (2.25-18.52)	0.002	2.08 (0.99-3.89)	< 0.001	0.001
	2011	2021	0.35 (-5.73-1.77)	0.728			
Non-Hispanic American Indian/Alaska Native	-	-	-	-	-	-	-
Non-Hispanic Asian or Pacific Islander	2001	2018	-1.02 (-1.66-0.95)	0.156	-2.60 (-3.66--1.34)	0.022	< 0.001
	2018	2021	-11.05 (-18.53--3.18)	< 0.001			
Non-Hispanic Black	2001	2009	9.19 (7.11-18.86)	0.002	3.01 (2.27-4.22)	< 0.001	0.005
	2009	2015	3.03 (-0.73-6.27)	0.081			
	2015	2021	-4.70 (-9.10--2.57)	0.007			
Non-Hispanic White	2001	2008	7.13 (4.84-10.18)	< 0.001	3.67 (3.44-4.00)	< 0.001	
	2008	2014	4.96 (3.67-8.98)	< 0.001			
	2014	2018	0.45 (-0.24-5.46)	0.133			
	2018	2021	-2.27 (-3.82--0.57)	< 0.001			
**Division 6:** East South Central (Alabama, Kentucky, Mississippi, Tennessee)							
Hispanic (All Races)	-	-	-	-	-	-	-
Non-Hispanic American Indian/Alaska Native	-	-	-	-	-	-	-
Non-Hispanic Asian or Pacific Islander	-	-	-	-	-	-	-
Non-Hispanic Black	2001	2013	7.40 (5.81-10.49)	< 0.001	3.39 (2.33-4.74)	< 0.001	0.01
	2013	2021	-2.33 (-6.31--0.03)	0.048			
Non-Hispanic White	2001	2007	9.67 (8.61-11.49)	< 0.001	5.09 (4.88-5.38)	< 0.001	
	2007	2014	6.07 (5.17-6.77)	< 0.001			
	2014	2021	0.39 (-0.24-0.95)	0.162			

Supplementary Table S1 (continued). Trends in HCC incidence rates among US Population by US Census Divisions and Race/Ethnicity, 2001 to 2021 (2020 skipped)

Population	Joinpoint Segment Year Start	Joinpoint Segment Year End	APC (95% CI)	*P*	Year 2001-2021 AAPC (95% CI)	*P*	Parallel Pairwise Comparison P-Value[a,b]
**Division 7:** West South Central (Arkansas, Louisiana, Oklahoma, Texas)							
Hispanic (All Races)	2001	2017	2.94 (2.58-3.48)	< 0.001	1.93 (1.59-2.31)	< 0.001	< 0.001
	2017	2021	-2.00 (-4.64-0.06)	0.059			
Non-Hispanic American Indian/Alaska Native	-	-	-	-	-	-	-
Non-Hispanic Asian or Pacific Islander	2001	2013	0.05 (-1.48-7.78)	0.723	-1.94 (-3.02--0.53)	0.02	< 0.001
	2013	2021	-4.85 (-10.68--2.44)	0.004			
Non-Hispanic Black	2001	2004	12.61 (8.09-20.43)	< 0.001	3.31 (2.84-3.85)	< 0.001	< 0.001
	2004	2014	4.65 (2.27-5.96)	0.003			
	2014	2018	-0.44 (-1.65-6.45)	0.941			
	2018	2021	-4.63 (-6.66--1.85)	< 0.001			
Non-Hispanic White	2001	2014	5.42 (4.75-6.66)	< 0.001	3.98 (3.43-4.56)	< 0.001	
	2014	2021	1.37 (-1.52-2.84)	0.158			
**Division 8:** Mountain (Arizona, Colorado, Idaho, Montana, Nevada, New Mexico, Utah, Wyoming)							
Hispanic (All Races)	2001	2014	3.96 (2.99-5.62)	< 0.001	1.38 (0.64-2.19)	0.002	0.006
	2014	2021	-3.25 (-6.55--1.22)	0.002			
Non-Hispanic American Indian/Alaska Native	-	-	-	-	-	-	-
Non-Hispanic Asian or Pacific Islander	2001	2003	23.36 (4.60-47.17)	0.03	-1.69 (-2.98--0.02)	0.048	0.001
	2003	2006	-13.83 (-18.92--3.78)	0.025			
	2006	2016	1.80 (0.16-11.02)	0.039			
	2016	2021	-9.38 (-16.33--5.20)	< 0.001			
Non-Hispanic Black	2001	2013	6.46 (3.41-17.38)	< 0.001	1.45 (-1.49-4.92)	0.169	0.025
	2013	2021	-5.64 (-19.28--0.77)	0.028			
Non-Hispanic White	2001	2016	4.29 (3.71-5.11)	< 0.001	2.42 (1.84-2.99)	< 0.001	
	2016	2021	-2.98 (-6.96--0.60)	0.016			
**Division 9:** Pacific (Alaska, California, Hawaii, Oregon, Washington)							
Hispanic (All Races)	2001	2013	3.54 (2.62-5.11)	< 0.001	1.39 (0.80-2.08)	< 0.001	0.002
	2013	2021	-1.74 (-4.05--0.35)	0.016			
Non-Hispanic American Indian/Alaska Native	2001	2021	3.92 (2.03-6.60)	< 0.001	3.92 (2.03-6.60)	< 0.001	0.028
Non-Hispanic Asian or Pacific Islander	2001	2007	0.67 (-1.08-6.00)	0.339	-2.32 (-2.81--1.82)	< 0.001	< 0.001
	2007	2015	-2.23 (-4.03--0.62)	0.039			
	2015	2021	-5.32 (-8.56--3.63)	< 0.001			
Non-Hispanic Black	2001	2009	4.79 (3.33-8.65)	< 0.001	-0.35 (-1.15-0.39)	0.36	< 0.001
	2009	2017	-0.27 (-2.04-1.30)	0.702			
	2017	2021	-10.02 (-15.90--6.73)	< 0.001			
Non-Hispanic White	2001	2013	5.47 (4.67-6.61)	< 0.001	2.15 (1.64-2.68)	< 0.001	
	2013	2021	-2.62 (-4.43--1.27)	0.001			

[a]A significant p-value indicates that the trends were not parallel (i.e. parallelism was rejected).
[b]Non-Hispanic White was used as reference group
Empty cells with a dash indicate suppressed data due to small sample size
